# Robust antibacterial activity of functionalized carbon nanotube- levofloxacine conjugate based on in vitro and in vivo studies

**DOI:** 10.1038/s41598-022-14206-w

**Published:** 2022-06-16

**Authors:** Marzieh Hassani, Azar Tahghighi, Mahdi Rohani, Malak Hekmati, Maryam Ahmadian, Hassan Ahmadvand

**Affiliations:** 1 Medicinal Chemistry Laboratory, Clinical Research Department, Pasteur Institute of Iran, Tehran, Iran; 2 Department of Medical Biotechnology, Faculty of Medicine, Lorestan University of Medical Sciences, Khorramabad, Iran; 3 Department of Microbiology, Pasteur Institute of Iran, Tehran, Iran; 4 Department of Organic Chemistry, Faculty of Pharmaceutical Chemistry, Tehran Medical Sciences, Islamic Azad University, Tehran, Iran; 5 Department of Biostatistics, School of Paramedical Sciences, Shahid Beheshti University of Medical Sciences, Tehran, Iran; 6 Department of Biochemistry, Faculty of Medicine, Lorestan University of Medical Sciences, Khorramabad, Iran

**Keywords:** Drug delivery, Antimicrobials, Microbiology, Chemistry, Nanoscience and technology

## Abstract

A new nano-antibiotic was synthesized from the conjugation of multi-walled carbon nanotubes with levofloxacin (MWCNT-LVX) through covalent grafting of drug with surface-modified carbon nanotubes in order to achieve an effective, safe, fast-acting nano-drug with the minimal side effects. This study is the first report on the evaluation of in vitro cell viability and antibacterial activity of nano-antibiotic along in addition to the in vivo antibacterial activity in a burn wound model. The drug-loading and release profile at different pH levels was determined using an ultraviolet–visible spectrometer. MWCNT-LVX was synthesized by a simple, reproducible and cost-effective method for the first time and characterized using various techniques, such as scanning electron microscope, transmission electron microscopy, and Brunauer–Emmett–Teller analysis, and so forth. The noncytotoxic nano-antibiotic showed more satisfactory in vitro antibacterial activity against *Staphylococcus aureus* compared to *Pseudomona aeruginosa*. The novel synthetic nano-drug possessed high loading capacity and pH-sensitive release profile; resultantly, it exhibited very potent bactericidal activity in a mouse *S. aureus* wound infection model compared to LVX. Based on the results, the antibacterial properties of the drug enhanced after conjugating with surface-modified MWCNTs. The nano-antibiotic has great industrialization potential for the simple route of synthesis, no toxicity, proper drug loading and release, low effective dose, and strong activity against wound infections. In virtue of unique properties, MWCNTs can serve as a controlled release and delivery system for drugs. The easy penetration to biological membranes and barriers can also increase the drug delivery at lower doses compared to the main drug alone, which can lead to the reduction of its side effects. Hence, MWCNTs can be considered a promising nano-carrier of LVX in the treatment of skin infections.

## Introduction

The introduction of antibiotics induced a radical decline in the rate of morbidity and mortality at the beginning of the twentieth century^1^. However, inappropriate and excessive use of antibiotics caused the prevalence of antibiotic tolerance and resistance in the past decades^2^. The global crisis of antimicrobial resistance currently threatens both the effective prevention and treatment of a wide range of bacterial infectious diseases^3^. With no effective antibiotics, the medical world can once again return to the “pre-antibiotic era". Consequently, the production of novel antibacterial agents or the application of innovative strategies to improve the pharmacokinetic and reduce resistance to available antibacterial drugs is of paramount importance.

Discovering new drugs is time-consuming and laborious. However, enhancing the efficacy of the existing antibacterial agents can accelerate the process of drug development through different techniques such as the combination therapies or the application of nanotechnology-based drug delivery systems^4,5^. The nanomaterials have attracted particular interest from various research groups due to their ideal physical and chemical properties, proper drug targeting efficiency, enhanced uptake, and suitable bio-distribution^5^. Recently, the application of nano-carriers has aroused considerable attention as the main strategy for improving antibacterial drug delivery, especially for the treatment of resistant infections^6^.

Carbon nanotubes (CNTs) are known as one of the most promising nano-carriers in biomedical applications due to their unique intrinsic properties, including hollow structure, high surface area to volume ratios, easy surface modification, ideal compatibility, and remarkable cell membrane penetration^7,8^. However, the hydrophobic nature, poor dispersibility, and toxicity effects of multi-walled carbon nanotubes (MWCNTs) can limit their applications in biomedical studies^9^. In order to overcome these drawbacks, the surface chemistry can be modified using different functionalization approaches and also the dispersity and biocompatibility need to be enhanced under physiological conditions^9,10^. Moreover, the decreased toxicity of the functionalized MWCNTs has been confirmed in previous research studies^4,11^.

Oxidation of MWCNTs with strong acids is known to be among the most promising and frequently employed approaches for surface modification^10^. This technique produces the carboxylic acid-functionalized MWCNTs (MWCNT-COOH) as efficient nano-platforms to immobilize multiple molecules by covalent bonds, hydrogen bonds, or π-π stacking interactions^12,13^. The functionalized MWCNTs with large surface areas possess a high loading capacity for various ligands such as peptides, proteins, nucleic acids, and drugs^14^. Moreover, they can be taken up by various tissues and cells without causing any damage, and depending on the types of surface functional groups, the loaded ligands can also be transferred^15^. The cellular internalization of CNTs contains three different mechanisms: endocytosis, phagocytosis, and direct translocation across the plasma membrane^15^. Studies have affirmed the effects of coating on the surface of CNTs in their internalization into mammalian cells^14,15^.

The chemically modified MWCNTs have been also extensively studied for their various biological activities, including antifungal, antibacterial, antiviral, and potent anticancer activities^16–18^. The crisis of both antibiotic tolerance and resistance encouraged many researchers to investigate antibacterial drug delivery systems by MWCNTs^19–22^. Also, the evaluation of the antibacterial effects of the carriers themselves (MWCNTs) and their functionalized forms was noticed by research groups.

A number of investigations have confirmed that the functionalization of MWCNTs improves their dispersion in aqueous media, resulting in the enhanced antibacterial activity, e.g. functionalizing with amino acids (arginine and lysine)^23^ and surfactants (polysorbates, sodium dodecylbenzene sulfonate, and hexadecyltrimethylammonium bromide)^24,25^.

Azizi-Lalabadi et al. have reviewed the antimicrobial activity of carbon nanomaterials and reported that MWCNTs with the surface factors (–OH and –COOH) do not display any bacteriostatic properties^26^. However, some studies have presented the proper bacteriostatic effects of the composites, including MWCNTs and silver nanoparticles against *S. aureu*s, *P. aeruginosa* and *Escherichia coli*, which can related to a powerful synergistic effect between MWCNTs and silver nanoparticles^26^. They also introduced the low-density polyethylene-based nanocomposites containing MWCNTs with antimicrobial activity against *E. coli*^26^. Nonetheless, the study of Ding and coworkers displayed the higher antibacterial activity of individually dispersed oxidized MWCNTs compared to aggregated raw MWCNTs^27^. Their study confirmed the importance of dispersion of MWCNTs in the antibacterial effect of CNTs.

The antimicrobial activity of pristine and functionalized MWCNTs with mono-, di-, and triethanolamine (MEA, DEA, and TEA) against multiple bacterial species demonstrated the direct impact of functional group type on antimicrobial properties of mentioned nanotubes (MWCNT-TEA > MWCNT-DEA > MWCNT-MEA > pristine MWCNT)^28^. The results of these surveys are powerful evidence approving the key role of functional groups in antimicrobial activities of MWCNTs. The functionalization of MWCNTs with an antibiotic can also ameliorate its antibacterial activity. Spizzirri et al. have synthesized bioconjugates using gelatin, MWCNTs, and fluoroquinolones and demonstrated that the hybrid nanomaterials greatly enhance antimicrobial activities against *Klebsiella pneumoniae* and *E. coli*^29^.

Selecting a suitable antibiotic in the drug delivery system has particular importance and can promote antimicrobial activity by a suitable synergistic effect between drug and nano-carrier. Levofloxacin (LVX) is a broad-spectrum, third-generation fluoroquinolone antibiotic with demonstrated activity against Gram-positive, Gram-negative, and anaerobic bacteria, which is mostly used for the treatment of many types of infections such as respiratory tract infections, post-inhalational anthrax, bacterial conjunctivitis, genitourinary infections, and skin and skin structure infections (SSSIs)^30,31^. SSSIs are an important issue in healthcare with a significant burden in infectious disease which is responsible to high rate of hospitalization. LVX with its once daily dose, great safety profile and easy transition to oral therapy is an proper selection to treat the full gamut of skin and skin structure infections.

Levofloxacin, as a safe and effective medicine, received FDA approval in the United States in 1996^31^. LVX inhibits bacterial DNA gyrase and topoisomerase IV, two critical enzymes for the transcription, replication, and repair of bacterial DNA^32^. This antibiotic is considered a preferable drug for the management of burn-associated infections^33^. However, due to its widespread application, resistant bacterial strains began to emerge under numerous clinical conditions^34^.

In developing countries, burns are recognized as one of the most common injuries with a high mortality rate^35^. Burns weaken the immune system and make the patient more susceptible to various infections as damaged skin cannot protect the body against microbes^36^. Therefore, a proper wound care is very important for reducing the risk of infection. The combination therapy with systemic and topical antibiotics, in comparison to systemic antibiotics alone, is more effective in the prevention of wound infection. Notably, topical antibiotics have the advantage of delivering high concentrations of antibiotics to the affected area, whereas systemic antibiotics need adsorption and then distribution throughout the body^37^. Due to blood vessel damage, systemic antibiotics cannot pass through the bloodstream and penetrate burn wounds^38^. Hence, the application of topical antibiotics is more common than systemic antibiotics^39^.

**Figure 1:**
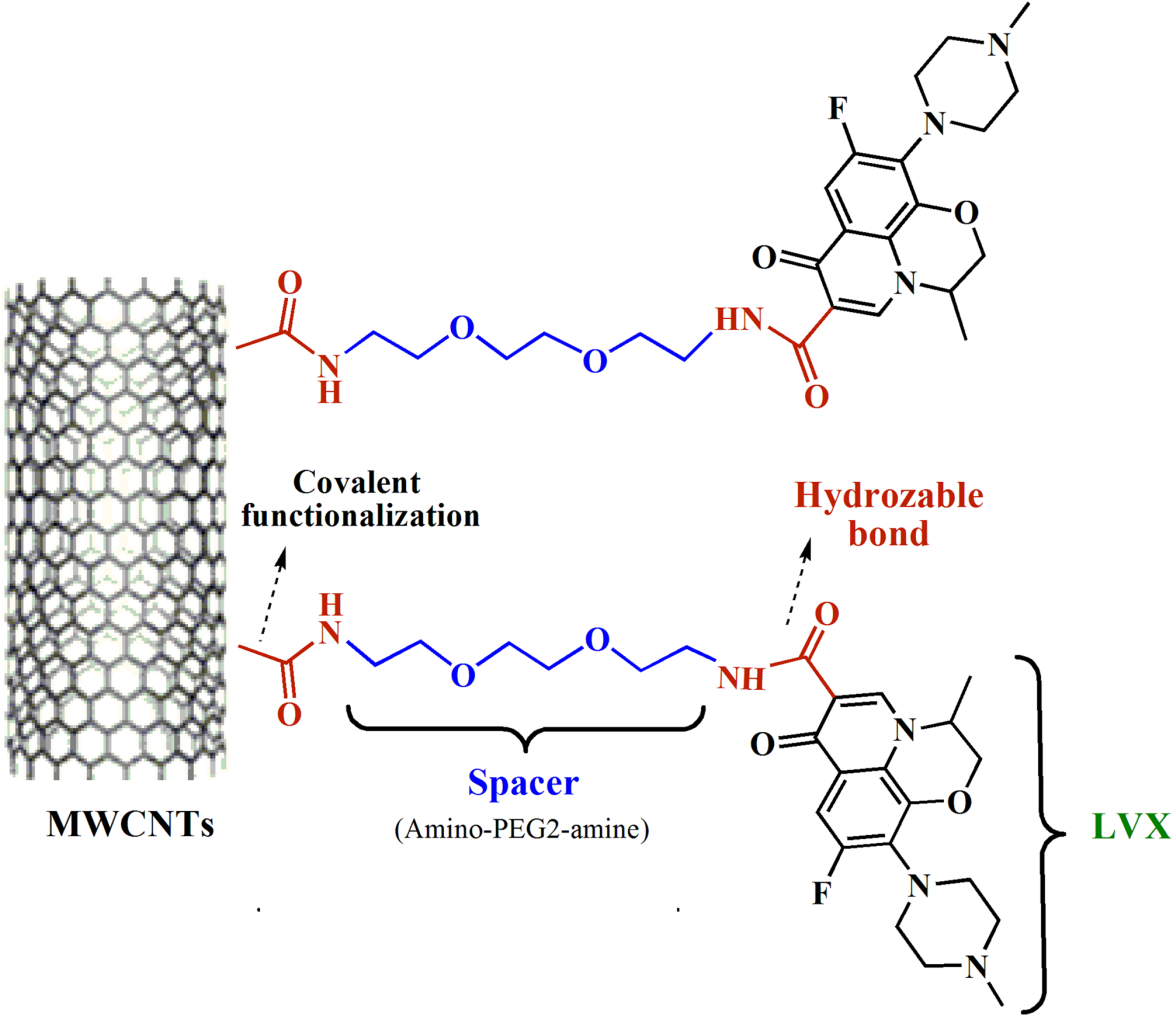
The covalent functionalization of MWCNTs with amino-PEG2-amine linker and levofloxacin. Abbreviations: MWCNTs: Multi-walled carbon nanotubes; LVX: Levofloxacin; PEG: Polyethylene glycol.

The current innovative study aimed to design and synthesize a new nano-antibiotic based on the covalent functionalization of MWCNTs with levofloxacin. For the enhancement of the aqueous dispersity and biocompatibility of nano-antibiotics, MWCNTs were connected to LVX by the amino-PEG2-amine linker (Fig. 1). The in vitro loading, release profile, and in vitro antibacterial activity of nano-drug against *S. aureus* and *P. aeruginosa* were evaluated, as well. Moreover, the cytotoxicity of nano-antibiotic was assessed on the fibroblast cell line using the MTT assay. In the end, the topical delivery of the antibiotic against the *S. aureus* skin infection mouse model was examined.

## Results

### Fourier-transform infrared (FTIR) spectroscopy

**Figure 2:**
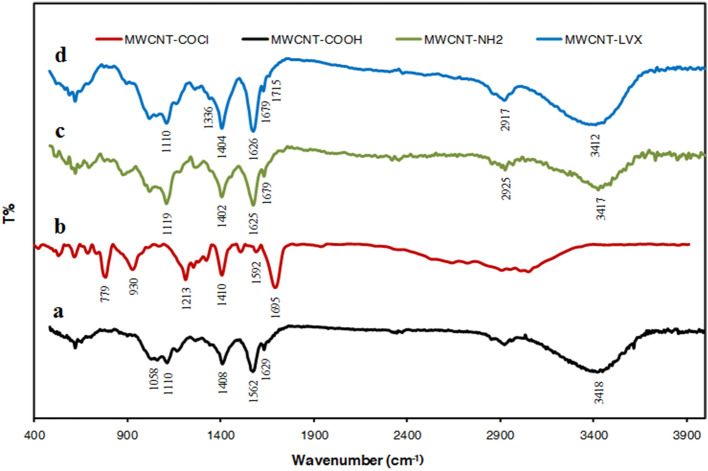
FTIR spectra of the samples: (**a**) MWCNT-COOH, (**b**) MWCNT-COCl, (**c**) MWCNT-NH_2_, and (**d**) MWCNT-LVX. Abbreviations: MWCNT: Multi-walled carbon nanotube; LVX: Levofloxacin; MWCNT-COOH: Carboxylic acid-functionalized MWCNTs; MWCNT-COCl: Acyl chloride-functionalized MWCNT; MWCNT-NH_2_: Amine-functionalized MWCNTs; MWCNT-LVX: Levofloxacin-loaded functionalized MWCNTs.

Figure 2 exhibits the FTIR spectra of all samples. Figure 2a demonstrates a strong band at 1629 cm^−1^ that was assigned to the C=O stretching vibrations in MWCNT-COOH. Accordingly, this could be attributed to the presence of carbonyl groups and was a proof of the successful oxidation of carbon nanotubes. The absorption bands at 1695 and 779 cm^−1^ could be ascribed to the C=O and C–Cl stretching vibrations, respectively, in MWCNT-COCl (Fig. 2b), which validated the successful chlorination of MWCNT-COOH. After the amidation reaction (Fig. 2c), the carbonyl stretching vibrations in MWCNT-NH_2_ appeared at 1625 and 1679 cm^−1^, which confirmed the elimination of acyl chloride and the formation of amide group (–CONH). Based on Fig. 2c and d, the bands in the range of 3417 and 3412 cm^−1^ were related to the stretching vibration of the primary and secondary amines in MWCNT-NH_2_ and MWCNT-LVX, respectively. The peaks at 3412, 1626, and 1110 cm^−1^ were respectively associated with the N–H, C=O, and C–N stretching vibrations in MWCNT-LVX. The peak at 2917 cm^−1^ corresponded to the methylene group from the polyethylene glycols (PEG)-linker. All FTIR findings validated the successful functionalization of MWCNTs.

The MWCNT-LVX data were compared with the standard spectrum of LVX and MWCNT-NH_2_ carrier, and their changes were noticed. Briefly, the bands of LVX were reported as follows: 3268 cm^−1^ (OH), 2959–2803 cm^−1^ (CH_2_, CH_3_), 1722 cm^−1^ (C=O acid, stretching vibration of the COOH), 1622 cm^−1^ (C=O ring), and 839 cm^−1^ (C–F). Aromatic hydrocarbons showed absorptions in the regions 1585 and 1404 cm^−1^^40^. The observation of a relative increase in the carbonyl band’s intensity at 1626 and 1679 cm^−1^ confirms the formation of new amide bonds, MWCNT-NH_2_ and LVX. Their interaction was also resulted in a broad band at 3412 cm^−1^ (3100–3600 cm^−1^ region), which can be related to –OH stretching vibration of LVX and –NH– stretching vibration of amide groups. The existence of a weak bond at 1715 cm^−1^ can be assigned to the carbonyl band of LVX. The mentioned peaks (3412 and 1715 cm^−-1^) in drug-loaded formulation may be related to the non-covalent interaction of the drug with the nano-carrier (MWCNT-NH_2_). Out-of-plane wagging at 650 cm^-1^ is the characteristic of primary amines.

### Raman analysis and X-ray diffraction (XRD) patterns

**Figure 3:**
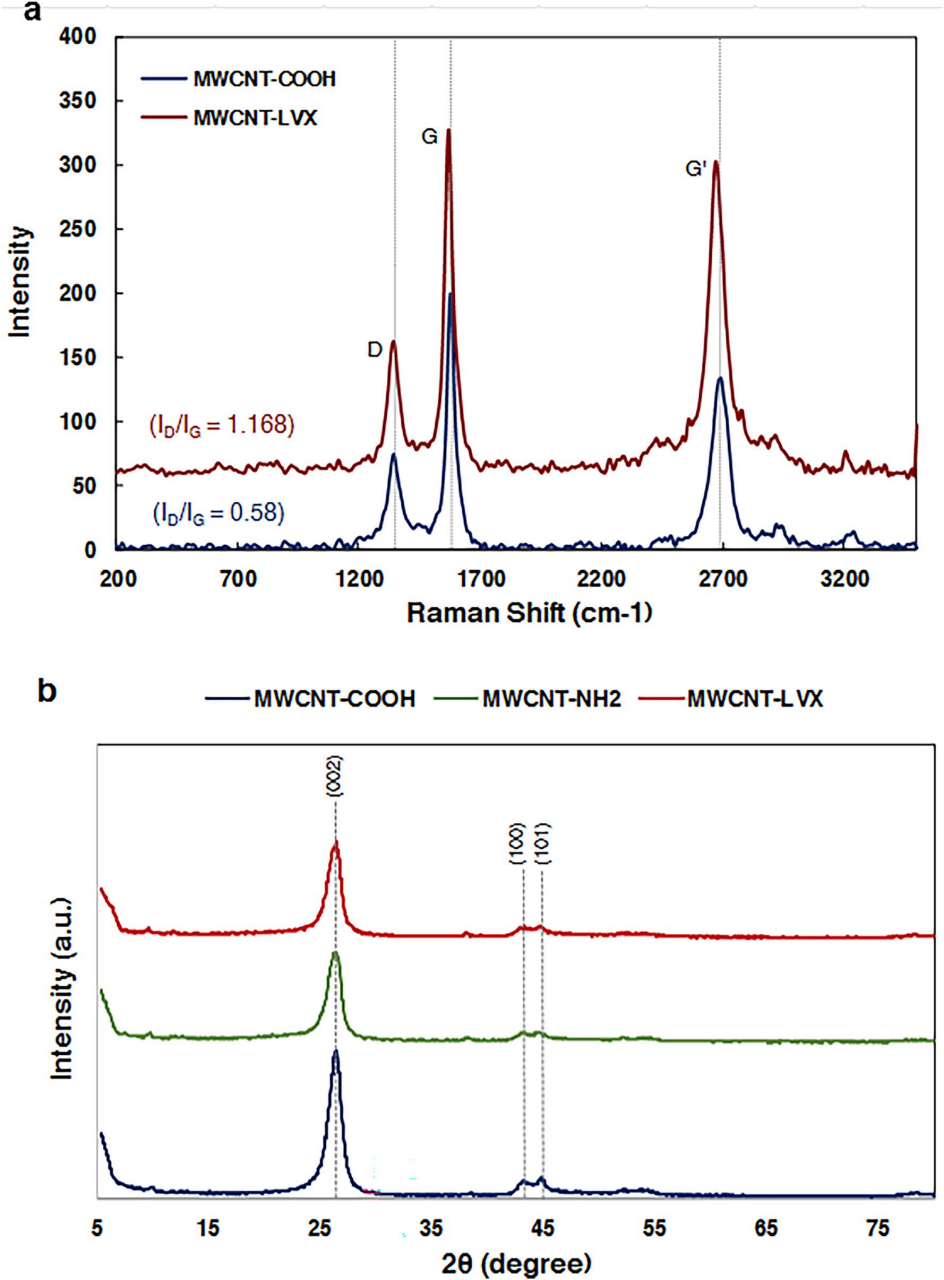
(**a**) Raman spectroscopy of MWCNT-COOH, and MWCNT-LVX; (**b**) XRD pattern of MWCNT-COOH, MWCNT-NH_2_, and MWCNT-LVX. Abbreviations: MWCNT: Multi-walled carbon nanotube; LVX: Levofloxacin; MWCNT-COOH: Carboxylic acid-functionalized MWCNTs; MWCNT-NH_2_: Aamine-functionalized MWCNTs; MWCNT-LVX: Levofloxacin-loaded functionalized MWCNTs.

Figure 3a shows the Raman spectra of MWCNT-COOH and MWCNT-LVX. Two main peaks in the Raman spectra were appeared at 1344.20 and 1570.97 cm^−1^, known as D and G bonds, respectively. The D band is related to disordered carbon atoms of MWCNTs corresponding to SP^3^ hybridization, and the G band shows the SP^2^ hybridization of carbon atoms in the graphene sheets. Area ratio of the D and G bonds (I_D_/I_G_) can be used to assess the amount of defects in the nanoparticle structure. I_D_/I_G_ ratio increased for MWCNT-LVX (I_D_/I_G_ = 1.168), which affirms the successful conversion of MWCNT-COOH to MWCNT-LVX. In the absence of amorphous carbon, the increase of I_D_ is related to the elevation of carbon containing SP^3^ hybridized and implies the successful functionalization reaction.

Figure 3b displays XRD spectroscopy patterns of MWCNT-COOH, MWCNT-NH_2_, and MWCNT-LVX. The XRD patterns of the oxidized CNTs and the functionalized CNTs have diffraction peaks at corresponding positions, indicating that MWCNT-NH_2_ and MWCNT-LVX still have the same tubular structure compared to MWCNT-COOH without any change in the lattice spacing. The intense peak at θ = 26° is indexed as the (002) reflection of the hexagonal graphite structure and the peak around 43° is resulted to the (100) graphitic planes. The XRD pattern clearly confirms the formation of nano-carrier (MWCNT-NH_2_) due to decreased intensity of peaks. The diffraction pattern of MWCNT-LVX showed the broad peak at 26° was deconvoluted into two peaks; the first peak centered at 2θ = 26.04°, and the other peak centered at 26.34°, clearly confirms the formation of nano-drug.

### Field emission scanning electron microscopy (FE-SEM)

**Figure 4:**
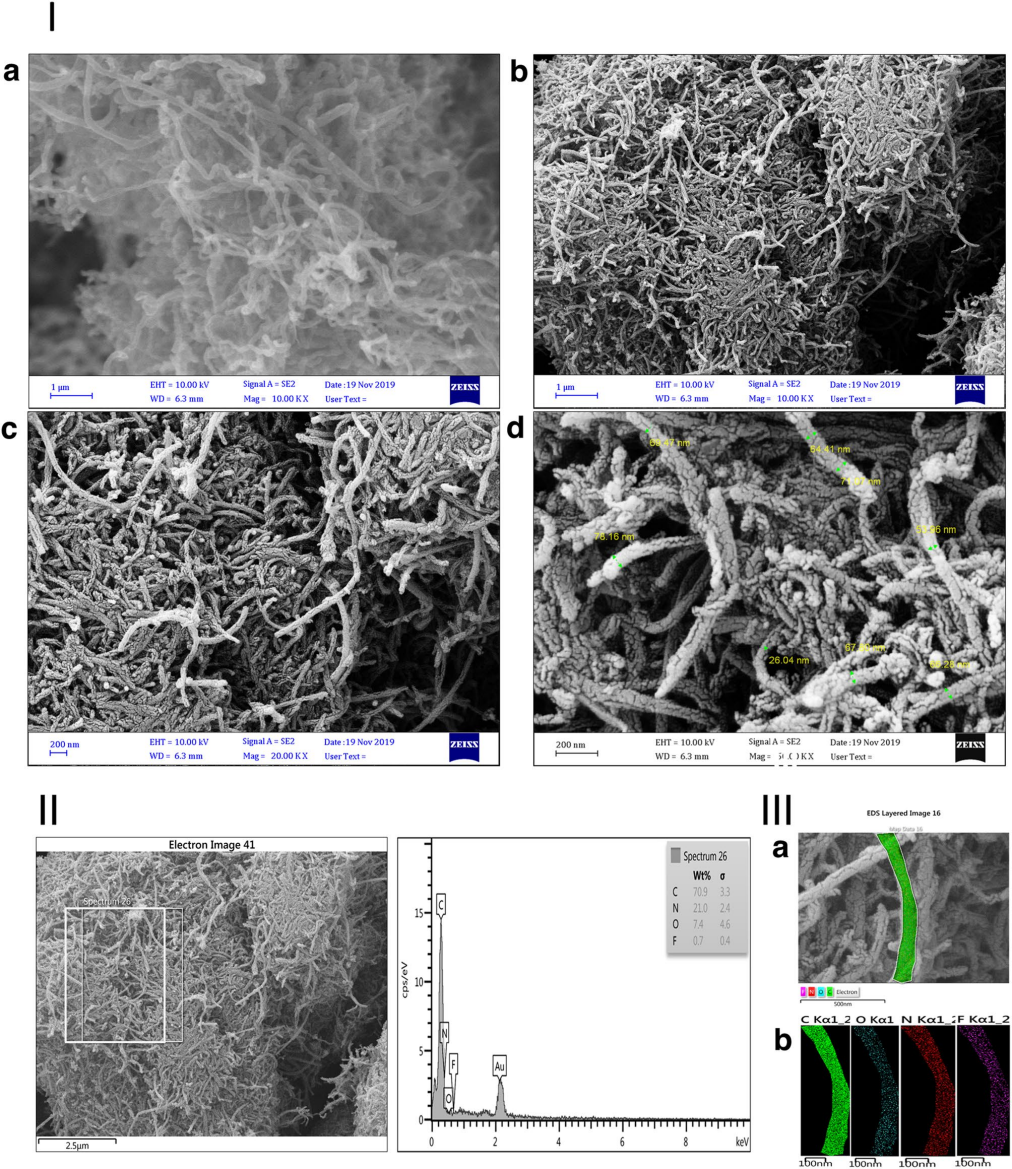
(**I**) FE-SEM images: (**a**) MWCNT-COOH, and (**b**–**d**) MWCNT-LVX with different magnifications: (**b**) 1 μm; 10.00 KX, (**c**) 200 nm; 20.00 KX, (**d**) 200 nm; 50.00 KX. (**II**) EDX mappings of the MWCNT-LVX, showing the distribution of C, N, O and F elements. (**III**) (**a**) FE-SEM image of MWMWCNT-LVX mapping analysis, (**b**) corresponding elemental mapping of C, N, O, and F elements. Abbreviations: FE-SEM: Field emission scanning electron microscopy; MWCNT: Multi-walled carbon nanotube; LVX: Levofloxacin; MWCNT-COOH: Carboxylic acid-functionalized MWCNTs; MWCNT-LVX: Levofloxacin-loaded functionalized MWCNTs.

The surface morphology of the modified and functionalized MWCNTs was investigated using FE-SEM. The FE-SEM image of MWCNT-COOH indicated its smooth, long, tortuous, and agglomerated structures (Fig. 4-Ia). However, the MWCNT-LVX showed a lower degree of entanglement and agglomeration in addition to rugged appearances compared to MWCNT-LVX. This observation is indicative of defect creation in nanotubes wherein the functional groups and drug were loaded, resulting in the appearance of irregular and branched sites on the MWCNTs surface (Fig. 4-Ib and Ic). Figure 4-Id validated fully coated surfaces, good dispersion, and well-organized assembly of MWCNTs-LVX.

### Energy dispersive X-ray spectroscopy (EDX)

EDX analysis was performed to quantify the components of MWCNT-LVX. In Fig. 4-II, EDX revealed strong signals for carbon (C) and small fractions of oxygen (O), which might be due to the acidic groups on MWCNT-COOH. The presence of both nitrogen (N) and fluorine (F) signals could also be attributed to amino-PEG2-amine linker and LVX and confirmed successful drug loading. Additionally, qualitative information about the distribution of various chemical elements in the nano-drug is presented in Fig. 4-IIIa and b. Thereafter, the elemental map analysis revealed a good dispersion of C, N, O, and F atoms on the surface of MWCNT-LVX.

**Figure 5:**
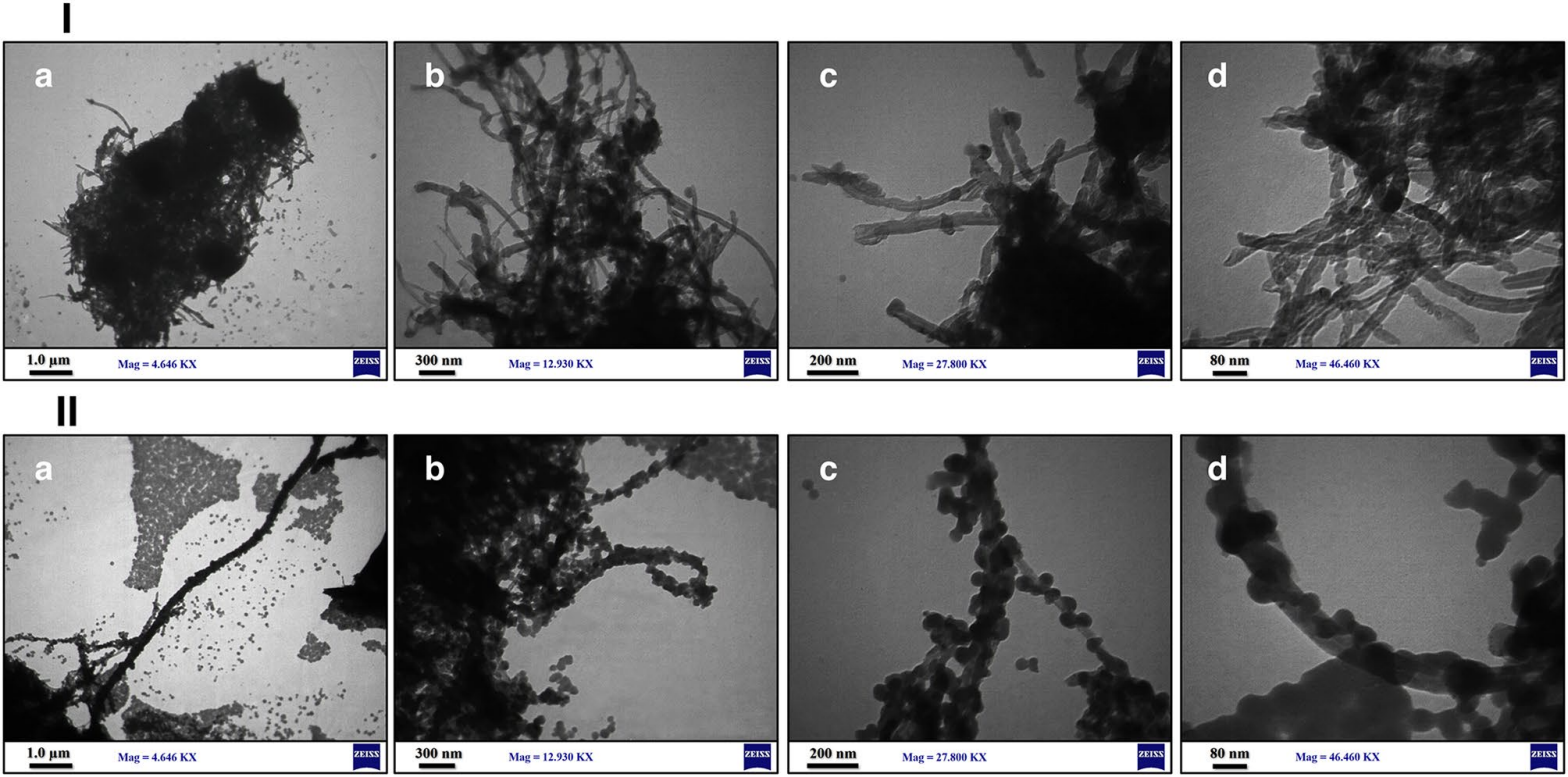
TEM image of the samples: (**I**) MWCNT-COOH, and (**II**) MWCNT-LVX with different magnifications (nm). Abbreviations: TEM: Transmission electron microscopy; MWCNT: Multi-walled carbon nanotube; LVX: Levofloxacin; MWCNT-COOH: Carboxylic acid-functionalized MWCNTs; MWCNT-LVX: Levofloxacin-loaded functionalized MWCNTs.

**Figure 6:**
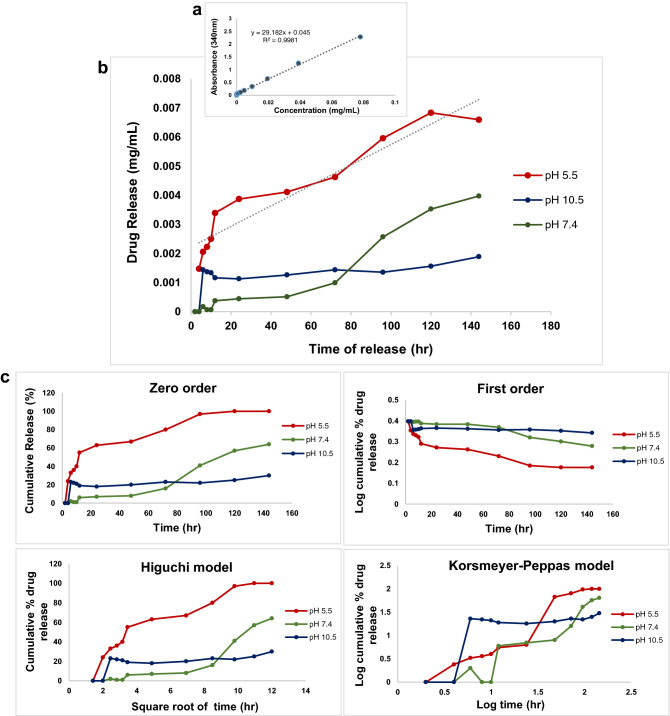
(**a**) Standard curve, absorbance at 290 nm vs. various concentrations of levofloxacin detected with UV–vis spectrometer; (**b**) pH responsive levofloxacin release profile of MWCNT-LVX within 144 h at various pHs (5.5, 7.4, and 10.5); (**c**) Drug release data fitted to various kinetic models (zero order, first order, Higuchi , and Korsmeyer–Peppas) obtained within 144 h at various pHs (5.5, 7.4, and 10.5). Abbreviations: MWCNT: Multi-walled carbon nanotube; LVX: Levofloxacin; MWCNT-LVX: Levofloxacin-loaded functionalized MWCNTs.

**Figure 7:**
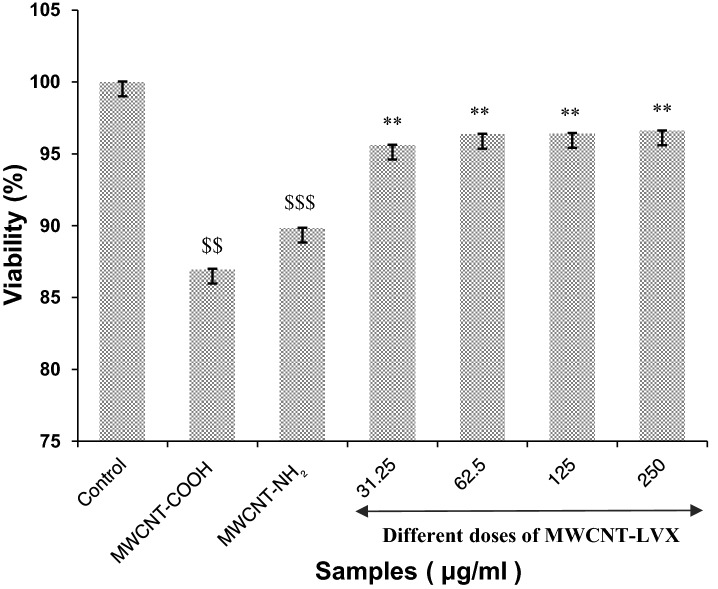
Effects of different doses of MWCNT-LVX, MWCNT-NH_2_, and MWCNT-COOH on cell viability. Data are shown as mean ± standard deviation from six separate experiments (n = 6). * and $ indicate statistically significant differences in cell viability. All deviations were taken as statistically significant if *p* < 0.05 and designated as **p < 0.01 vs. CNT-NH_2_ group; $$ *p* < 0.01 and $$$ *p* < 0.001 vs. control group. Abbreviations: MWCNT: Multi-walled carbon nanotube; LVX: Levofloxacin; MWCNT-COOH: Carboxylic acid-functionalized MWCNTs; MWCNT-NH_2_: Amine-functionalized MWCNTs; MWCNT-LVX: Levofloxacin-loaded functionalized MWCNTs.

**Figure 8:**
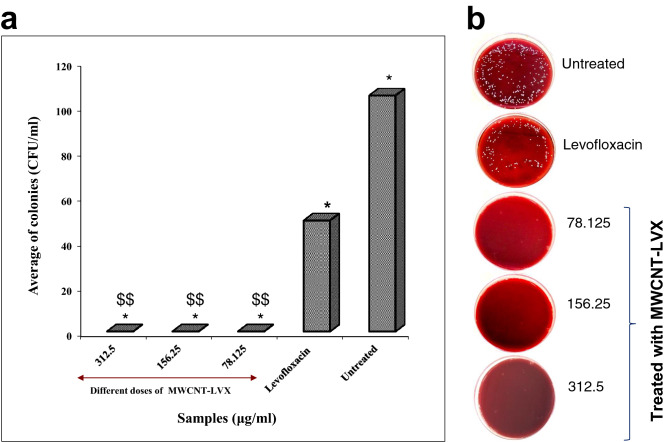
(**a**) All deviations were taken as statistically significant if *p* < 0.05 and designated as * *p* < 0.05 and $$ *p* < 0.01 vs. untreated group. (**b**) Colonies grown on blood culture medium; a dilution of 10^6^ CFU/mL was used for each sample (the lowest dilution). As evident from the Figure, the MWCNT-LVX samples have greatly inhibited the survival of *S. aureus* in comparison to the controls (Levofloxacin-treated and untreated samples). Abbreviations: MWCNT: Multi-walled carbon nanotube; LVX: Levofloxacin; MWCNT-LVX: Levofloxacin-loaded functionalized MWCNTs.

**Figure 9:**
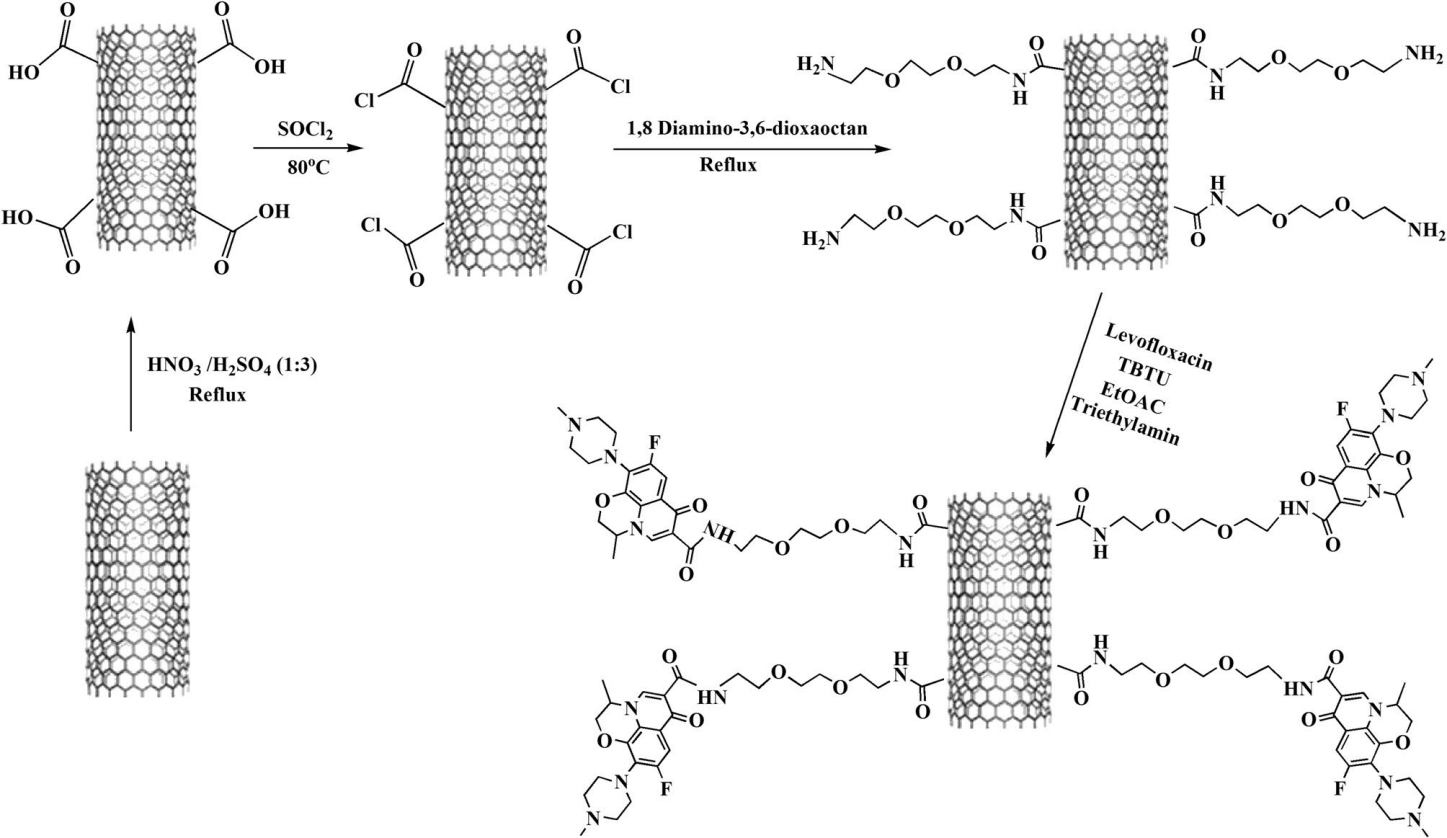
Nano-drug synthesis diagram.

### Transmission electron microscopy (TEM)

The morphologies and distributions of MWCNTs were investigated using TEM technique. TEM images (Fig. 5-Ia-d) verified the tubular morphology of acid-treated MWCNTs, as well as the smooth and homogeneous surface. As shown in Fig. 5-Ia, at magnification of 1 μM, MWCNT-COOH has large agglomerates. Figure 5-IIa, b, c, and d show the TEM images of MWCNT-LVX with different magnifications (1000, 300, 200, and 80 nm, respectively). The adhesion of LVX (shown with black dots in Fig. 5-IIc and d) is probably occurred by various interactions, including non-covalent interactions (π-π stacking and hydrogen bonds) with CNTs and covalent grafting with amine groups on MWCNTs-NH_2_. As shown in Fig. 5-IIa, at magnification of 1 μM, MWCNT-LVX has been completely dispersed. TEM images confirmed the structural integrity and a suitable distribution of LVX in the coated MWCNT-LVX.

### Brunauer–Emmett–Teller (BET) surface area analysis

In this study, the BET method was applied to describe the specific surface area based on the nitrogen adsorption–desorption isotherms. The BET surface areas, the total volume, and the mean diameter of the oxidized MWCNTs and PEGylated MWCNTs loaded with LVX were given in Table 1. The study of the porous structure of samples showed the decreased pore diameter, pore volume, and surface area in MWCNT-LVX compared to MWCNT-COOH. The surface area, and pore volume play a key role in the adsorption of different bioactive molecules^41^. As can be seen in Table 1, MWCNT-COOH has a surface area of 73.033 m^2^/g, and its total pore volume is 1.015 cm^3^/g. It was found that the BET surface area and the total pore volume of the MWCNT-LVX synthesized by the coupling of the modified oxidized MWCNTs (MWCNT-NH_2_) with LVX decreased, which may be due to a change in its morphology. These results proved the successful drug loading on MWCNT-COOH.

**Table 1:** BET surface areas, pore diameter, and pore volumes of the oxidized and drug-loaded multi-walled carbon nanotubes.

Sample	Surface area (m^2^/g)	Total pore volume (cm^3^/g)	Mean pore diameter (nm)
MWCNT-COOH	71.033	1.015	57.183
MWCNT-LVX	57.732	0.677	46.877

Abbreviations: BET: Brunauer, Emmett and Teller, MWCNT: Multi-walled carbon nanotube, LVX: Levofloxacin, MWCNT-COOH: Carboxylic acid-functionalized MWCNTs, MWCNT-LVX: Levofloxacin-loaded functionalized MWCNTs.

### CHN elemental analysis

As tabulated in Table 2, the high percentages of H and N atoms in MWCNT-NH_2_ were related to the correct functionalization of MWCNT-COOH. Additionally, increased nitrogen content in MWCNT-NH_2_ confirmed a successful amidation reaction. The high percentages of carbon, hydrogen, and nitrogen contents in MWCNT-LVX could arise from the conjugation of LVX with amine-modified MWCNTs. Resultantly, the successful synthesis of nano-antibiotic could be validated.

**Table 2:** Elemental analysis values relative to oxidized, amidated and drug-loaded multi-walled carbon nanotubes (MWCNTs).

Element	MWCNT-COOH	MWCNT-NH_2_	MWCNT-LVX
Carbon	77.86	66.98	90.32
Hydrogen	0.24	0.65	0.97
Nitrogen	0.18	1.2	2.48

Abbreviations: MWCNT: Multi-walled carbon nanotube, LVX: Levofloxacin, MWCNT-COOH: Carboxylic acid-functionalized MWCNTs, MWCNT-NH_2_: Amine-functionalized MWCNTs, MWCNT-LVX: Levofloxacin-loaded functionalized MWCNTs.

### Drug loading, in vitro drug release, and release kinetics mechanism

The standard curve of LVX solution is shown in Fig. 6a. The drug entrapment of 90% and loading efficiency (LE) of 80% were reported for MWCNT-LVX. These results demonstrated the great loading capacity of MWCNTs. LVX could most probably bind to nanotubes by π-π stacking, hydrophobic-hydrophobic, hydrogen bonding, and covalent interactions.

As depicted in Fig. 6b, the release profile of LVX in phosphate buffer solutions (PBS) with different pH values confirmed a sustained drug release capacity of MWCNT-LVX at pH 5.5, while the release rate stayed nearly constant throughout the study and increased with a mild slope. Nano-antibiotic had a slow-release at pH 7.4 before 72 h; however, the release rapidly increased between 72 to 120 h. This phenomenon indicates that pH 7.4 is not suitable for drug delivery, as compared to pH 5.5. Furthermore, the curve at pH 10.5 demonstrated no efficient release.

The in vitro drug release profile of MWCNT-LVX in various pH medias was plotted according to four kinetic equations (including the zeroth-order, first order, Higuchi and Korsmeyer-Peppas), and the results are presented in Fig. 6c and Table 3^42^. Korsmeyer-Peppas model showed the best fit and highest correlation at pH 5.5. The value of n factor in this model reflects the possible release mechanisms of drug according to super case-II transport, which affirms the surface loss as a controlling factor of drug release. However, the kinetic analysis of drug release profile in neutral media showed that the drug-delivery system predominantly released LVX in a first-order manner with R^2^ value of 0.9418 (Table 3). This kinetics states that alteration in concentration regarding time change is dependent on only concentration. Kinetics release in pH 10.5 did not follow any model.

**Table 3:** Drug release kinetics of levofloxacin from nano-drug formulation at various pH media (5.5, 7.4, and 10.5).

Model									
pH	Zero order		First order		Higuchi		Korsmeyer-Peppas		
	R^2^	K_0_(h^-1^)	R^2^	K_1_(h^-1^)	R^2^	K_H_ (h^-1/2^)	R^2^	K_kp_	n
Acidic	0.8127	3.0117	0.8609	0.0894	0.9101	10.6350	0.9491	45.290	1.1861
Neutral	0.9301	0.2546	0.9418	0.0964	0.8527	1.8693	0.8876	6.9323	1.0685
Basic	0.3653	1.0292	0.3766	0.1161	0.4331	3.8042	0.4670	7.850	0.5738

*R*^*2*^ Determination coefficient, *k*_*0*_ Zero-order rate, *k*_*1*_ First-order rate, *K*_*H*_ Higuchi release rate, *K*_*KP*_ Korsmeyer–Peppas release rate, *n* n factor in Korsmayer–Peppas model.

After an in vitro drug release test, the FTIR spectra of all dried samples were again recorded and confirmed the differences in the functional groups in MWCNT-LVX at pH 5.5 after seven days (unpublished data). This result was in line with ultraviolet–visible (UV–Vis) data, though the least differences were obtained at pH 10.5.

### In vitro antibacterial tests

As can be seen in Table 4, the antibacterial effect of MWCNT-LVX on *S. aureus* at acidic pH was more appropriate than neutral and alkaline media. All wells corresponding to MWCNT-NH_2_ and MWCNT-COOH were turbid, indicating no inhibition of bacterial growth. Meanwhile, a minimum bactericidal concentration (MBC) test revealed no bacterial growth for MWCNT-LVX at pH 5.5, which confirmed the minimum inhibitory concentration (MIC) results (Table 4). The control wells confirmed the complete bacterial growth at various pHs.

**Table 4:** The MICs of samples at different pH and times and the MBCs (μg/mL) at different pHs.

Bacterial strains	Samples	pH 5.5			pH 7.4			pH 10.5		
		MIC (μg/mL)		MBC (μg/mL)	MIC (μg/mL)		MBC (μg/mL)	MIC (μg/mL)		MBC (μg/mL)
		36 h	18 h		36 h	18 h		36 h	18 h	
*S. aureus* (ATCC 25,923)	MWCNT-LVX	19.531	39.062	78.125	39.062	78.125	156.25	78.125	78.125	312.5
	MWCNT-NH_2_	–	–	–	–	–	–	–	–	–
	MWCNT-COOH	–	–	–	–	–	–	–	–	–
	LVX	0.488	0.488	0.488	0.488	0.244	0.488	0.244	0.244	0.488
*P. aeruginosa* (ATCC 27,853)	MWCNT-LVX	625	1250	1250	625	1250	1250	1250	1250	2500
	MWCNT-NH_2_	–	–	–	–	–	–	–	–	–
	MWCNT-COOH	–	–	–	–	–	–	–	–	–
	LVX	0.976	1.953	1.953	1.953	1.953	1.953	3.906	3.906	3.906

Abbreviations: MIC: Minimum inhibitory concentration, MBC: Minimum bactericidal concentration, MWCNT: Multi-walled carbon nanotube, LVX: Levofloxacin, MWCNT-COOH: Carboxylic acid-functionalized MWCNT, MWCNT-NH_2_: Amine-functionalized MWCNT, MWCNT-LVX: Levofloxacine-loaded functionalized MWCNT.

### MTT assay

As shown in Fig. 7, nano-carriers (MWCNT-NH_2_ with 89.83% and MWCNT-COOH with 86.98% viability) manifested very low toxicity in their highest dose. The percentages of viable cells increased in cell lines exposed to MWCNT-LVX (cell viability > 95%) relative to the amidated and oxidized CNTs, which can be justified by proper surface modifications in MWCNT- LVX conjugate. Cell viability at different concentrations of MWCNT-LVX (31.25, 62.50, 125, and 250 μg/mL) was in the narrow range of 95.61–96.60%. These results confirmed the noncytotoxic effect of nano-antibiotic.

### In vivo therapeutic efficacy of MWCNT-LVX

Figure 8 presents the bactericidal effects of MWCNT-LVX on a burn infection model caused by *S. aureus*. Accordingly, MWCNT-LVX at 312.5, 156.25, and 78.125 μg/mL concentrations showed no growth of bacterial colonies throughout the treatment. This outcome could be attributed to the efficiency of the drug delivery system in MWCNT-LVX compared to the LVX-treated group (Fig. 8a). The bacterial growth inhibition is more noticeable in the images of Petri disks (Fig. 8b). Statistical analysis showed that MWCNT-LVX (at all three doses) significantly suppressed the bacterial count in the infected burn wound compared to the control group (*p* < 0.02). In addition, the untreated group (the negative control group) had the highest number of colonies, while the treated group with LVX (the positive control group) showed fewer colonies. Moreover, in the untreated group, increase in the numbers of colonies was significant compared to the groups treated with MWCNT-LVX (*p* < 0.005; Fig. 8a). As tabulated in Table 5, the numbers of colonies are given in mean ± standard deviation of CFU/mL for each skin sample.

**Table 5:** Number of colonies grown on culture medium.

Groups	Mean ± SD
MWCNT-LVX (312.5 μg /mL)	0
MWCNT-LVX (156.25 μg /mL)	0
MWCNT-LVX (78.125 μg /mL)	0
Levofloxacin (positive control)	49.2 × 10^6^ ± 18.4 × 10^6^
Untreated (negative control)	104.8 × 10^6^ ± 88.8 × 10^6^

Abbreviations: MWCNT: Multi-walled carbon nanotube, LVX: Levofloxacin, MWCNT-LVX: Levofloxacine-loaded functionalized MWCNTs, SD: Standard deviation.

## Discussion

The current study aimed to design and synthesize a new nano-antibiotic based on the covalent functionalization of MWCNTs with LVX by the formation of the amide bonds. LVX, a broad-spectrum antibiotic, is administered systemically for the treatment of patients with uncomplicated skin or skin structure infections, including burn injury^43^. According to the guidelines of each country, 500–750 mg of oral or intravenous LVX once daily for 7–14 days is recommended^44^. This interesting candidate has several side effects, such as phototoxicity, gastrointestinal disturbances, peripheral neuropathy, hepatotoxicity, tendinitis, and stimulation of the central nervous system^45,46^. On the other hand, its dose adjustment is crucial in patients with impaired renal function.The topical administration of LVX can eliminate its adverse effects by decreasing required dose as a function of drug concentration in the site of infection, as well as by minimizing the first-pass effect.

In the past, invasive burn wound infections would have easily led to sepsis and death^47^. Although the introduction of antimicrobials played an essential role in reducing the mortality rate, burn infections still can lead to a significant delay in wound healing, formation of deep scars, rejection of skin graft, prolonged hospitalization, and increased economical costs^48^. Early burn wound infections are related to Gram-positive bacteria, especially staphylococci that is located in hair follicles and sweat glands that strongly colonize the wound surface within the first 48 hours^49,50^. Gram-negative bacteria, like *P. aeruginosa* from an environmental source and/or the patient’s endogenous gastrointestinal flora, is considered as another important cause of burn wound infections^49^. Accordingly, both of them produce many virulence factors that are significant in the pathogenesis of invasive infections. However, the most common causative pathogens in complicated skin infections is *S. aureus* but not *P. aeruginosa*, with the prevalence of 46 and 11%, respectively^33^.

Systemic and topical antibiotic therapies are effective in the prevention and treatment of wound infections. The failure of systemic antibiotics to prevent and treat burn wound infections is frequently described by the avascular nature of the eschar, the thickness of eschar, and the early extension of burn wounds^38,51^. On the other side, systemic antibiotics are widely related to resistance mechanisms, which can consequently endanger the treatment process^52^. Therefore, the antimicrobials are often administered topically in the forms of ointment, cream, dressings, or solutions; accordingly, they can contribute to a high and sustained concentration of the drug at the site of the infection due to its increased skin permeability^53,54^. Despite the potential benefits of topical antibiotic therapy, the sub-optimal outcomes are still unexploited^55^. Thus the necessity of innovation in the topical drug delivery field is amplified. For this purpose, a variety of new nano-drug delivery systems has been introduced^56^. An ideal nano-carrier for topical drug administration should be non-toxic and biodegradable with drug controlled and sustained release ability^57^. In order to achieve maximum efficiency in nano-delivery systems, the proper drug-candidate selection is also of paramount importance. Nano-formulation of LVX has previously been considered by scientists for its broad-spectrum activity against various pathogens, its easy entrance to the infected sites, low dosing, and tolerable toxicity^58^. LVX is a hydrophilic drug that can be rapidly absorbed within the first 2 hours^59^, and its integration into the nano-carriers can lead to a slower release rate, thus enhancing activity.

The prepared nano-formulations of LVX could improve its activity against various bacterial pathogens like *S. aureus*^58–62^. Levofloxacin-loaded PLGA particles presented drug LEs of ~ 70% with a sustained release profile at pH 7.4 for up to 4–6 weeks, which inhibited the biofilm formation of *S. aureus* and deteriorated established biofilm^58^. According to the study of Montanari and coworkers, the incorporation of LVX into self-assembled hyaluronan-cholesterol nanohydrogels (LVX-NHs) displayed an activity similar to free LVX against extracellular *S. aureus*^60^. However, the intracellular antibacterial activity of LVX-NHs greatly enhanced by this drug delivery system in *S. aureus*-infected keratinocytes. In the same study, %50 release of LVX was observed in LVX-NHs over 5 h^60^. In a study conducted by Valizadeh et al. (2021), a sesame oil nanoemulsion containing LVX was prepared, which showed some beneficial effects on various phases of the wound healing process in the diabetic ulcers of a *S. aureus* model^61^.

Currently, CNTs are broadly applied for the target-specific drug delivery of various pharmaceutical ingredients^62,63^. CNTs possess a unique feature due to their facile transport through cellular membranes^64^. The conjugation of drugs with CNTs can ultimately facilitate its transfer into bacterial cells, which then leads to high local drug concentration^65^. Siafaka et al. (2019) prepared various LVX-loaded nanomaterials and reported LVX-loaded MWCNT had an antibacterial activity similar to LVX against *S. aureus*^22^*.* They identified a direct interaction between LVX and pristine MWCNTs by van der Waals forces with low drug loading (10.1%) and 40 and 100% sustained release after 2 and 24 h, respectively^22^. According to the mentioned studies^22,58–61^, various nano-carriers showed a range of medium to good results for the drug delivery of LVX to bacterial infections. Indeed, the selection of a suitable functional group in the nano-formulations of drugs has a crucial role and directly affects their biological activity and physico/chemical properties^66^.

Polyethylene glycols usually act as one of the most important linkers between MWCNT-COOH and ligands due to their great biocompatibility and wide applications in biomedical areas, especially drug delivery systems^13,14^. The PEGylation of CNTs increases their dispersity and cellular uptake, while diminishing their toxicity. Therefore, in the present study, MWCNT-LVX conjugate was synthesized based on the covalent functionalization of PEGylated MWCNTs (MWCNT-NH_2_) with LVX (Fig. 1). The large surface area of MWCNTs permitted extremely high drug loading efficiency by various interactions of LVX on the inner and outer surface of PEGylated MWCNTs (MWCNT-NH_2_) to construct a new nano-drug formulation, namely, MWCNT–LVX (Fig. 5). The BET surface area analysis could also confirm this claim (Table 1). Most likely, the aromatic rings in LVX enhanced its interaction with the polyaromatic surface of MWCNTs via hydrophobic and π–π stacking interactions^67^. Hydrogen-bonding also played a significant role in the adsorption of drug by CNTs with interaction between complementary hydrogen-bonding donor and hydrogen-bonding acceptor moieties^68^. Additionally, the covalent attachment of LVX to the nanotubes walls occurred through the amidation of MWCNT-NH_2_ with carboxylic group of LVX; consequently, a high drug loading (LE = 80%) and a considerable decrease in toxicity compared to free LVX was observed.

Notably, the backbone amide linker strategy in drug delivery systems, as a great method, is mostly used to release the active medications in the tissues, organs, and cells, as documented in literature^69,70^. Indeed, various proteolytic enzymes can slowly hydrolyze protease‐sensitive linkers, such as amide bonds, and then release drugs over a long period of time^70^. In the skin microbiome, commensal and pathogenic species mainly secrete proteases^71^. The secreted proteases from skin commensals participate in homeostatic bacterial coexistence, while pathogenic bacterial proteases are applied as virulence factors, which consequently lead to the breached integrity of the epithelial layer. Regardless of their profits and losses, these enzymes can hydrolyze amide or peptide bonds and then assist in releasing the drug from its nano-formulation on the skin.

Drug release behavior is a crucial factor in drug delivery systems. Actually, the sustained release of antimicrobial agents can help to prevent wound infection and facilitate wound healing by enhancing high local concentrations of drugs and reducing systemic toxicity^72^.

There have been some evidences about the relation between pH and wound healing^73,74^. Healthy skin has an acidic pH, which after burn injury, it rises to an alkaline pH. Increasing pH values in the wound surface and exudates after the burn decrease the number of normal bacterial flora but increase the number of varied species of pathogenic bacteria in the wound surface, which consequently leading to the impairment of wound healing. Some bacteria also produce ammonia, which in itself is necrotizing, and which can damage tissue oxygenation by increasing the pH^74^.

In burn wounds infected by *S. aureus*, the pH of wound exudate increases prior to the onset of clinical signs of local burn infection. It has also been identified that antibiotic activity against methicillin-resistant *S. aureus* enhances when the pH value of culture plate diminishes to 5.5 compared with a pH 7.0^75^. Therefore, performing a rapid treatment with the continuous release of antibiotics can keep the acidic pH range and accelerate the wound healing process.

In our study, pH-controlled LVX delivery using MWCNT-LVX at pHs 5.5, 7.4, and 10.5 showed a pH-triggered response. A sustained and complete drug release in a gradual manner was obtained at pH 5.5 for MWCNT-LVX until the fifth day (Fig. 6b). As shown in Fig. 6b, the 6 and 24 h release of LVX were found to be ~ 25 and 50% in acid media (pH 5.5). LVX was also released in a slow and controlled manner at pH 7.4 after the fourth day, which was significantly less than at pH 5.5. Indeed, the noncovalent attachment of LVX to the surface-engineered MWCNTs involves hydrogen bonds. The hydrogen bonding interactions are weakened at low pH (5.5) and a higher amount of drug can be released^76^. On the other hand, the amide bonds between LVX and MWCNT-NH_2_ are probably hydrolyzed steadily in addition to proteolytic processes in acidic media, and the drug could be released over a long time. Based on the results, likely, none of these release processes have occurred in basic media (pH 10.5; Fig. 6b). It can be concluded that MWCNTs enable the delivery of bioactive agents in a pH-triggered and/or pH-responsive manner. Besides, a single dose of nano-antibiotic (MWCNT-LVX) can maintain high local drug concentration for several days and reduces multiple dose administration. The reduced dose assists in the prevention of adverse side effects and diminishes the healthcare costs associated with poisonings, which was also validated by Daughton et al.^77^.

CNTs tend to agglomerate, making their dispersion problematic in biological media. However, in the current study, MWCNT-LVX was easily dispersed in different solutions compared to CNT-NH_2_ and CNT-COOH. Perhaps, the covalent grafting of LVX on nanotube surfaces offers a wide menu for surface functionalization along with steric stabilization^78^.

The morphological study (Figs. 4 and 5) represented the loading of drug on the external and internal surfaces of MWCNTs, while the nano-drug had preserved their tubular shapes even after the coating process with drug. In general, The FE-SEM images (Fig. 4) showed different surface morphology of MWCNT-LVX compared to MWCNT-COOH. The rough and groovy surface of nano-antibiotic, compared to the smooth surface of MWCNT-COOH, might be due to the place of the drug on the surface functionalized MWCNTs by its non-covalent and covalent attachments. Considering the level of magnification achieved, FE-SEM did not show the proper dispersion of the functionalized nanotube ropes. However, TEM with high magnification could affirm the dispersion of the functionalized MWNTs, supporting the assumption that nanotube ropes via functionalization were well separated. According to the TEM analysis (Fig. 5), the long and hollow tubular structures of the oxidized MWCNTs (MWCNT-COOH) were visible and interlocked with each other (Fig. 5I). However, after the coating of the modified MWCNTs (MWCNT-NH_2_) with respective drug, a layer of the drug loaded on the MWCNT surfaces was observed, when compared to the uncoated MWCNT-COOH (Fig. 5II).

The internal cavities of MWCNTs provided high drug loading capacity. As seen in Fig. 5I and II, in contrast to unloaded MWCNT-COOH, the hollow tubes were blocked in MWCNT-LVX by some particles, indicating the loading of drugs into these cavities. This phenomenon could be justified by π-π stacking, hydrophobic-hydrophobic, hydrogen bonding, and covalent interactions of LVX on the external and internal surfaces of MWCNTs. The FE-SEM and TEM images demonstrated that the coating is relatively uniform.

The possible toxicity of CNTs remains another major concern in biomedical applications. However, in this regard, Kolosnjaj-Tabi et al. (2010) reported the safety of oral CNTs in a dose of up to 1000 mg/kg^79^. The available safety data also confirmed the low toxicity of CNTs through various exposure pathways^80^. Indeed, several parameters such as size, length, dispersibility, functionalization, administration doses, and trace metal contaminants can affect the toxicity of CNTs^80,81^. In the present study, the cytotoxicity results proved that PEGylated nanotube coating with LVX could tremendously improve the biocompatibility of MWCNT-LVX in comparison to MWCNT-NH_2_ and MWCNT-COOH.

Functionalization of pristine MWCNTs with carboxyl and amino groups led to diminishing MWCNTs toxicity^82,83^. These functionalized MWCNTs, however, exhibited a stronger tendency to form big agglomerates in protein-rich biological media^82^. In our study, for the preparation of the stock suspensions, the method of sonication was performed to disperse the samples in a cell culture medium, in which the nano-drug could just sufficiently be dispersed. The formation of large agglomerates of carboxylated and PEGylated-functionalized MWCNTs can be related to their increased toxicity compared to MWCNT-LVX.

In this study, the synthetic nano-antibiotic showed a suitable in vitro bactericidal activity against *S. aureus* in comparison with *P. aeruginosa* at different pH levels (Table 4). The MWCNT-LVX also indicated more satisfactory results against *S. aureus* at acidic pH, similar to the skin environment. Excellent result was observed in an in vivo study when the nano-antibiotic solution was topically administered on *S. aureus*-infected burn wounds.

The bacterium *S. aureus* is able to adapt rapidly to host cells, especially immune cells, in order to increase its survival in unsuitable conditions, which in turn can lead to the failure in infection treatment. In our study, MWCNT-LVX disrupted *S. aureus* survival more effectively than LVX alone, which might be related to high drug loading and the slow release of LVX on the skin’s acid mantle. Indeed, fresh burn wounds with acidic pH resulted in a sustained release of LVX and conclusively potent activity against *S. aureus*. In addition, there is emerging evidence of the internalization of MWCNTs in bacteria cells^65,84,85^. LVX exerts its antimicrobial activity via the inhibition of type II topoisomerases and ultimately through the inhibition of bacterial DNA replication against *S. aureus* and *P. aeruginosa*. The conjugation of LVX with MWCNTs can facilitate its transfer into bacterial cells by its internalization, giving rise to the high local concentration of LVX and its rapid bactericidal activity.

No skin damage or inflammation was observed in wounds treated with MWCNT-LVX, not even in the deepest layers, confirming the high safety of the new nano-antibiotic. Based on the results, we can speculate that if MWCNT-LVX is absorbed through the skin to the systemic blood circulation, it does not have any toxicity at its low effective dose. The results of the present study, including high drug loading, proper release profile, nontoxicity and great in vivo antibacterial activity, showed the superiority of our study over other studies surveyed the antibacterial activity of some nano-formulations of LVX^58–62^.

However, future studies will be needed to evaluate the antibacterial effects of this nano-drug on other pathogens to confirm its high efficacy. Additionally, other studies are required to be performed to determine the safety, pharmacokinetics, and the ultimate fate of this new nano-antibiotic in the human body.

## Conclusion

An antibacterial nano-conjugate was successfully synthesized based on the covalent grafting of modified multi-walled CNTs with LVX, which is the first report on this conjugation. Amino-PEG2-amine linker between MWCNT and drug enhanced the aqueous dispersity and biocompatibility of this nano-antibiotic. High drug loading and the pH-sensitive release of LVX with a suitable antibacterial activity in acidic media were observed, which affirmed MWCNT-NH_2_ as a promising drug delivery tool for antibacterial agents. A potent antibacterial activity was also found following the topical administration of the nano-antibiotic solution on *S. aureus*-infected burn wounds. This new nano-antibiotic with improved biocompatibility and no toxicity can be a potential candidate for the control and treatment of wound infections induced by *S. aureus* as a topical drug delivery system. Despite numerous surveys and the hopeful results on drug nano-formulations for the therapeutic management of infections, few formulations have been offered for clinical trials so far. In the field of drug delivery research, achieving an efficient delivery system with high safety, low cost, and easy manufacturing is an desirable goal. According to the results of this study, MWCNT-LVX with a simple route of preparation, no toxicity, high drug loading capacity, low effective dose, and potent activity against wound infections possess high industrialization potential. However, further studies are needed to be conducted to place such nano-formulations in the pharmaceutical market.

## Materials and methods

### Materials and instrument

MWCNTs (purity > 90%, mean diameter ~ 20–30 nm, length ~ 10–30 μm, SSA > 110 m^2^/g) were purchased from US Research Nanomaterials (USA). Nitric acid, hydrogen peroxide 30%, sulfuric acid, sodium hydroxide, Mueller Hinton agar (MHA), and Mueller Hinton broth (MHB) were purchased from Merck Company (Darmstadt, Germany). Thionyl chloride, levofloxacin, 1,8-diamino-3,6-dioxaoctane, triethylamine, tetrahydrofuran (THF), 2-(1*H*-benzotriazole-1-yl)-1,1,3,3-tetramethylaminium tetrafluoroborate (TBTU), and 3-(4,5-dimethylthiazol-2-yl)-2,5-diphenyltetrazolium bromide (MTT) were obtained from Sigma-Aldrich (Germany). Oxidized MWCNTs as ~ 6% w/w were prepared based on literature^86,87^.

A microplate reader (ELx808, BioTek, USA) was also applied to measure the absorbance. FT-IR spectra were recorded using a Perkin Elmer Spectrum Two FT-IR Spectrometer, USA. Raman measurements were performed using a XploRA plus Raman microscope with a 532 nm laser (Horiba, Japan). The XRD patterns were recorded on an X-ray diffractometer (PW1730, Philips, Netherlands) with Cuk α radiation (λ equal to 1.54056 Å) radiation.

FE-SEM (ZEISS-Sigma VP model, Germany) was applied to examine the morphology of the samples. An energy-dispersive X-ray (EDX, Oxford Instruments, UK) analysis was performed to determine the elemental composition of MWCNT- LVX. The distribution pattern of structural elements was also determined by elemental mapping images. Morphology and structural transformations of the samples were investigated by TEM (ZEISS-EM10C-100 kV model, Germany). The adsorption and desorption isotherms of nitrogen were measured using the BELSORP-mini II apparatus (MicrotracBEL, Japan). The elemental analysis (2400 series II CHNS elemental analyzer, Perkin-Elmer Co., USA) determined the carbon, hydrogen, and nitrogen contents.

### Ethical considerations

In this study, the in vivo experiments and animal care were approved by the Biomedical Research Ethics of Lorestan University of Medical Sciences (Ethics Code: IR.LUMS.REC.1397.199). All methods were carried out in accordance with the animal welfare guidelines and regulations^88^. All experiments were reported in conformity with ARRIVE guideline 2.0.

### Functionalization of MWCNTs

The functionalization of oxidized carbon nanotube (MWCNT-COOH) was performed according to a previous method with some modifications^89^. MWCNT-COOH (200 mg) along with excess thionyl chloride (30 mL) as a reagent and solvent were sonicated for 30 min within an ultrasonic bath, and then the reaction mixture was refluxed at 80 °C for 24 h. The obtained product, acyl chloride-functionalized MWCNT (MWCNT-COCl) was then filtered under vacuum by a 0.2 μm porous polytetrafluoroethylene (PTFE) membrane filter (Whatman) and washed with dry THF (3 × 50 mL) to remove the excess of thionyl chloride. The corresponding acyl chloride without further purification was immediately mixed with 1,8-diamino-3,6-dioxaoctane (2.5 mL) in 75 mL of dry THF and refluxed at 80 °C for 48 h to praper amine-functionalized MWCNT (MWCNT-NH_2_). Subsequently, the mixture was cooled to room temperature and filtered under vacuum on a 0.2 μm PTFE filter. Finally, the resulting precipitate (MWCNT-NH_2_) was washed with dry THF (3 × 50 mL) and then dried in a vacuum oven for 4 h at 50 °C (Fig. 9).

### Synthesis of nano-antibiotic (MWCNT-LVX)

A volume of 200 mg of LVX, 86 mg of TBTU, and trimethylamine (0.118 mL) in ethyl acetate (25 mL) were stirred under argon atmosphere for 1 h. Thereafter, 100 mg of MWCNT-NH_2_ was added to the mixture, sonicated for 1 h within an ultrasonic bath, magnetically stirred for 24 h at room temperature, and then filtered under vacuum on 0.2 μm PTFE filter. The solid product (MWCNT-LVX) was washed with ethyl acetate (3 × 50 mL) and methanol (3 × 50 mL) and then dried in a vacuum oven at 60 °C for 8 h^90^.

### Loading LVX on PEGylated MWCNTs

The calibration curve of LVX was plotted based on the maximum wavelength of (λ_Max_) 290 nm using a UV–Vis spectrophotometer (R^2^ = 0.9981, y = 29.182x + 0.045). Next, MWCNT-LVX and its blank sample (without loaded drug) were prepared similarly according to the synthesis procedure of nano-antibiotic as mentioned above. After 24 h, both the reaction mixtures were centrifuged at 2000 rpm (CMF 15KR, Tigra, Poland) for three times until the supernatant became colorless. The supernatant was filtered using a 0.2 μm PTFE membrane filter, and solid residues were then washed with ethyl acetate and methanol (50 × 3 mL each). Then the total volume of filtrate plus wash solutions were collected and measured. The absorbance of the solution was determined at 290 nm using a UV–Vis spectrophotometer (Cecil CE 1021, UK). Finally, the drug entrapment efficiency (EE) and LE were obtained using the following formulas^91^:

$$\% {\text{Entrapment}}\;{\text{efficiency}}\left( {\% {\text{EE}}} \right) = \frac{{\left[ {{\text{Drug}}} \right]{\text{total}} - \left[ {{\text{Drug}}} \right]{\text{supernant}}}}{{\left[ {{\text{Drug}}} \right]{\text{total}}}} \times 100$$

$$\% {\text{Entrapment}}\;{\text{efficiency}}\left( {\% {\text{LE}}} \right) = \frac{{{\text{Weight}}\;{\text{of}}\;{\text{loaded}}\;{\text{drug}}\;{\text{in}}\;{\text{MWCNTs}}}}{{{\text{Weight}}\;{\text{of}}\;{\text{total}}\;{\text{MWCNTs}}}}$$

### In vitro drug release test

To investigate the drug release of MWCNT-LVX under acidic, neutral, and basic environments, PBS was prepared at pHs 5.5, 7.4, and 10.5. Nano-drug (2.5 mg) was dispersed in dialysis bags (12–14 kDa MWCO, 23 mm flat width, Sigma-Aldrich) containing 1 mL of PBS. Accordingly, each dialysis bag was separately immersed in 50 mL of PBS (pHs 5.5, 7.4, 10.5) and then stirred at room temperature at a speed of 80 rpm. At various time intervals between 2 and 144 h, 1 mL of each sample was taken out to test LVX concentration at 290 nm (Cecil CE 1021, UK). After each sampling time, 1 mL of fresh buffer was replaced to maintain a constant initial volume^92^. The drug release kinetics were determined using specific mathematical models and plotted according to zero order (cumulative % of drug released vs. time), first order (log cumulative % of drug remain), Higuchi model (cumulative % of drug released vs. square root of time), and Korsmeyer–Peppas model (log cumulative % of drug released vs. log time) equations^93^.

### In vitro antimicrobial activity of MWCNT-LVX

The antibacterial activity of nano-antibiotic was determined using MIC and MBC tests according to Clinical Laboratory Standard Institute (CLSI)^94^. Two different strains of pathogenic bacteria, including *S. aureus* (ATCC 25,923) and *P. aeruginosa* (ATCC 27,853), were prepared from Microbial Collection (Pasteur Institute of Iran, Tehran) and cultured on blood agar at 37 °C overnight to obtain single and pure colonies. Subsequently, the optical density (OD) of bacterial suspensions were measured in the range of 600–625 nm using a UV–Vis spectrophotometer to attain 0.5 McFarland turbidity standards and then diluted (~ 10^6^ CFU/mL). The aqueous suspensions (10 mg/mL) of MWCNT-LVX, MWCNT-NH_2_, and MWCNT-COOH, in addition to LVX solution (1 mg/mL) were prepared. The test compounds (100 μL) were added to the first row of 96-well plates containing 100 μL of MHB, then mixed and transferred to the second row. This procedure was repeated till the last well, from which 100 μL was removed. Finally, 100 μL of the microbial suspension was added to all wells. After incubation at 35 °C for 18 and 36 h, the turbidity of the wells were assessed, and the MIC was defined as the lowest concentration with no visible bacterial growth. For the MBC test, 100 μL of the MIC well was streaked on the MHA plates and incubated at 35 °C for 18 h. The MBC value was defined as the lowest bactericidal concentration without any bacterial colony growth on the agar plate. These tests were separately performed for both strains at different pHs 5.5, 7.4, and 10.5 in triplicate.

### Cell viability assay

Cell viability was evaluated on mouse fibroblast cell line L929 (NCBI C161 was obtained from the National Cell Bank of Iran [NCBI], Pasteur Institute of Iran) using MTT colorimetric assay^95^. The cells were grown in RPMI 1640 medium (Gibco, Waltham, MA) containing 10% fetal bovine serum (FBS) and incubated in 90% humidified atmosphere with 5% CO_2_ at 37 °C. Briefly, 1 × 10^4^ cells/well in 100 μL of RPMI1640 medium were seeded onto 96-well tissue culture plates and incubated at 37 °C for 24 h, for the cells adhesion. Afterward, the culture medium was replaced with 90 μL of the samples (MWCNT-LVX [250, 125, 62.5, and 31.25 μg/mL]), MWCNT-NH_2_ [250 μg/mL], or MWCNT-COOH [250 μg/mL]) and 10 μL of FBS. The control wells contained only RPMI1640 and FBS. After 48 h, the supernatants were changed with 100 μL of MTT solution (0.5 mg/mL), and the plates were incubated at 37 °C for 4 h. Then, the reaction solutions were removed, and the formazan crystals dissolved in isopropanol (100 μL). The plate was finally incubated on a shaker for 15 min, and the absorbance was measured at 570 nm using a microplate reader. This experiment was performed in sextuplicate, and the cell viability was calculated using the following expression:

$$\% {\text{Viability}} = \left( {\frac{{{\text{mean}}\;{\text{OD}}\;{\text{of}}\;{\text{sample}}}}{{{\text{mean}}\;{\text{OD}}\;{\text{of}}\;{\text{control}}}}} \right) \times 100$$

### In vivo antimicrobial activity of MWCNT-LVX

A total of 25 female NMRI strain mice (aged almost eight weeks with bodyweight of about 30–35 g) were purchased from the Pasteur Institute of Iran and kept for one week under standard conditions (24 ± 2 °C and 52% humidity) with adequate food and water. Mice were randomly divided into five equal groups, including three treatment groups and two control groups. Afterward, the *S. aureus* (ATCC 25,923) suspensions (approximately 10^5^ CFU/mL) were cultured on blood agar. Mice were then anesthetized intraperitoneally [ketamine/xylazine, (5/1 mg/kg)]. Subsequently, their dorsal hair was shaved, cleaned, and disinfected with 70% (v/v) ethanol. Burn wounds (the second-degree) were created by a cylindrical metal rod (10 mm diameter, 50 g weight) which was heated to 100 °C and then pressed for 5 s on the dorsal thoracic region in the low part of the mouse body about 1 cm away from the vertebral column in the right side. The criteria to diagnosis of second degree burn were determined by major following indications including alteration of skin colour to brown, skin roughness and ruffling on the surrounding the burn wound. The injured mice were immediately placed in separate cages. After 1 h, all burn wounds were inoculated with 100 μL of the bacterial suspension (10^5^ CFU/mL) and treated with 100 μL of LVX (0.488 μg/mL, positive control) and different concentrations of MWCNT-LVX aqueous solutions (312.5, 156.25, and 78.125 μg/mL) at 1 h post-infection. MWCNT-LVX solutions were dispersed completely by sonication before topical administration. The volume of the whole solution was transferred to the wound in two steps (2 × 50 μL) without any wastage. The group with no treatment was considered as a negative control. After 24 h, the mice were humanely killed, and the burned skin lesions were removed using sterile surgicassors and homogenized in 1 mL of sterile PBS. The tissue samples were serially diluted six-fold, and then all six dilutions were cultured on blood agar plates. After incubation for 24 h at 37 °C, the number of colonies was counted, and the results were expressed as the mean ± standard deviation of CFU/mL per skin sample^96^.

### Statistical analysis

Statistical analysis was performed using SPSS software (version 22). Data were analyzed by one-way analysis of variance (ANOVA), Shapiro–Wilk and Kolmogorov–Smirnov tests followed by Tukey post-hoc test. Data were reported as a mean value with its standard deviation indicated (mean ± SD), and *p*-values ≤ 0.05 were considered statistically significant.
